# Dialyl-sulfide with trans-chalcone prevent breast cancer prohibiting SULT1E1 malregulations and oxidant-stress induced HIF1a-MMPs induction

**DOI:** 10.18632/genesandcancer.237

**Published:** 2024-08-09

**Authors:** Aarifa Nazmeen, Sayantani Maiti, Smarajit Maiti

**Affiliations:** ^1^Department of Biochemistry, Cell and Molecular Therapeutics Lab, Oriental Institute of Science and Technology, Midnapore 721101, India; ^2^Haldia Institute of Health Sciences, ICARE, Haldia, East Midnapore, India; ^3^AgriCure Biotech Research Society, Midnapore, WB, India

**Keywords:** SULT1E1, breast cancer, HIF1-a and MMPs, Dialyl-sulfide and chalcone, MMPs, therapy

## Abstract

Background: In some breast cancers, altered estrogen-sulfotransferase (SULT1E1) and its inactivation by oxidative-stress modifies E2 levels. Parallelly, hypoxia-inducible tissue-damaging factors (HIF1α) are induced. The proteins/genes expressions of these factors were verified in human-breast-cancer tissues. SULT1E1 inducing-drugs combinations were tested for their possible protective effects.

Methods: Matrix-metalloproteases (MMP2/9) activity and SULT1E1-HIF1α protein/gene expression (Western-blot/RTPCR) were assessed in breast-cancers versus adjacent-tissues. Oxidant-stress neutralizer, chalcone (trans-1,3-diaryl-2-propen-1-ones) and SULT1E1-inducer pure dialyl-sulfide (garlic; *Allium sativum*) were tested to prevent cancer causing factors in rat, *in-vitro* and *in-vivo*. The antioxidant-enzymes SOD1/catalase/GPx/LDH and matrix-degenerating MMP2/9 activities were assessed (gel-zymogram). Histoarchitecture (HE-staining) and tissue SULT1E1-localization (immuno-histochemistry) were screened. Extensive statistical-analysis were performed.

Results: Human cancer-tissue expresses higher SULT1E1, HIF1α protein/mRNA and lower LDH activity. Increase of MMP2/9 activities commenced tissue damage. However, chalcone and DAS significantly induced SULT1E1 gene/protein, suppressed HIF1α expression, MMP2/9 activities in rat tissues. Correlation and group statistics of t-test suggest significant link of oxidative-stress (MDA) with SULT1E1 (*p* = 0.006), HIF1α (*p* = 0.006) protein-expression. The non-protein-thiols showed negative correlation (*p* = 0.001) with HIF1α. These proteins and SULT1E1-mRNA expressions were significantly higher in tumor (*p* < 0.05). Correlation data suggest, SULT1E1 is correlated with non-protein-thiols.

Conclusions: Breast cancers associate with SULT1E1, HIF1α and MMPs deregulations. For the first time, we are revealing that advanced cancer tissue with elevated SULT1E1-protein may reactivate in a reducing-state initiated by chalcone, but remain dormant in an oxidative environment. Furthermore, increased SULT1E1 protein synthesis is caused by DAS-induced mRNA expression. The combined effects of the drugs might decrease MMPs and HIF1α expressions. Further studies are necessary.

## INTRODUCTION

Breast cancer is the most common female cancer globally, representing nearly a quarter (25%) of all cancers. Organic carcinogens covalently bind DNA forming stable and depurinating adducts. The compound 4-hydroxyestradiol and estradiol-3, 4-quinone (E1(E2)-3,4-Q)) produces significantly higher levels of depurinating adduct and smaller stable adduct [1]. These estrogen-DNA adducts are quickly lost by the cleavage of the glycosyl bond from DNA and create apurinic sites that can cause cancer [2, 3]. Mounting evidences demonstrate that a few E2 metabolites (i.e., CE-3, 4-Q) interact with DNA and form 4-OHE1(E2)−1-N3Ade and 4-OHE1(E2)-1-N7Gua that causes DNA mutation and potential cellular transformation [4, 5].

The use of human breast epithelial cell lines such as MCF-10F, an immortalized, non-transformed estrogen receptor (ER)-α-negative cell line has identified the initiation of cancer by estrogen-DNA adducts. Results indicate that beside the alteration of E2-ER signaling, genotoxic effects via estrogen metabolite are partly responsible for the malignant transition [6]. These findings support the hypothesis that the availability of the active estrogen is a critical determinant in the treatment of some breast cancer.

A large percentage of breast cancers are sensitive to estrogen and have a good response to endocrine therapy based on selective ER modulators (SERM) i.e., tamoxifen [7] and fulvestrant [8]. Another crucial strategy is the inhibition of aromatase, which reduces the synthesis of estrogen [9]. Beyond these strategies, estrogen metabolizing enzymes are given less priority for research. SULT1E1, a phase II metabolizing enzyme that is located in the cytoplasm and responsible for sulfonation of estrogen [10]. SULT1E1 polymorphisms are shown to be a risk factor for breast and endometrial cancers, [11] suggesting that its modulation might be an attractive strategy in the prevention, management and or treatment of breast cancer.

Some epidemiological data indicate reduced percentages of women in high-income nations are diagnosed with metastatic breast cancer. The majority of individuals diagnosed with distant metastatic stage had older ages and lower socioeconomic status [12]. The burden is not spread equitably and that there are significant differences in incidence, mortality, and survival between and within certain countries and regions. The population structure like age, race, and ethnicity, lifestyle, environment and other confounding factors contribute to these variances in global disease prevalence [13]. Regarding some molecular and genetic factors, a substantial enrichment in genes linked to several cellular functions and related activities was found by involved in estrogen related cancer [14]. It was concluded that the local estrogen metabolism may be a target for epithelial ovarian cancer (EOC) treatment. As previously mentioned, SULT1E1 is transcriptionally regulated under oxidative stress. Oxidative stress responses augment Nrf-2 and HIF-1a in breast cancer cell. Hypoxia Inducing Factor (HIF) target genes in every step of the metastatic process. Degeneration of extracellular matrix is implicated as the metastatic growth factor and MMPs are highly expressed in advanced breast cancer. A high expression of MMP-2 and MMP-9 was found in breast cancer patients [15]. So, it is suggested that the restriction in HIF1α and MMP activities may have therapeutic prospects against breast cancer.

In our earlier study, we explored that the pre-tumorigenic condition induced by ethylnitrosourea (ENU) and E2 showed impairment of SULT1E1 expression and E2 regulations via oxidant-stress signaling [16]. Thus, induction and activation of SULT1E1 might become cancer prevention and treatment strategy. Dialylsulfide (DAS) increase of SULT1E1 mRNA and protein in the liver of female mice. [17]. Despite of significant SULT1E1 induction by DAS, the endogenous E2 level was unaltered Contrarily, the clearance of exogenously administrated E2 was accelerated in DAS treated mice [18]. This suggests that under variable oxidative stress condition, SULT1E1 transits between gain and loss of enzyme activity. Our previous report suggests that oxidative stress induced SULT1E1 modifications may be similar in human breast cancer tissue and experimental animal model [16]. SULT1A1 also sulfoconjuagtes E2 but at higher concentration and contributes to control the E2 pool. Chalcone possesses oxidative stress relieving property and act as antioxidant in different diseases [19].

Under the purview of literature study, the objective of the current study was to explore the role of SULT1E1 and HIF1α proteins/genes expressions in human breast cancer tissues in relation to this disease severity. Further, we investigated the possible influence of di-allyl sulfide and chalcone on SULT1E1 and HIF1α proteins/genes expressions aiming to disease prevention strategy. Our present study may have some therapeutic implications in breast cancers.

## RESULTS

### Statistical analysis of relation between Cancer manifestation and its causative factors

**Figure 1:**
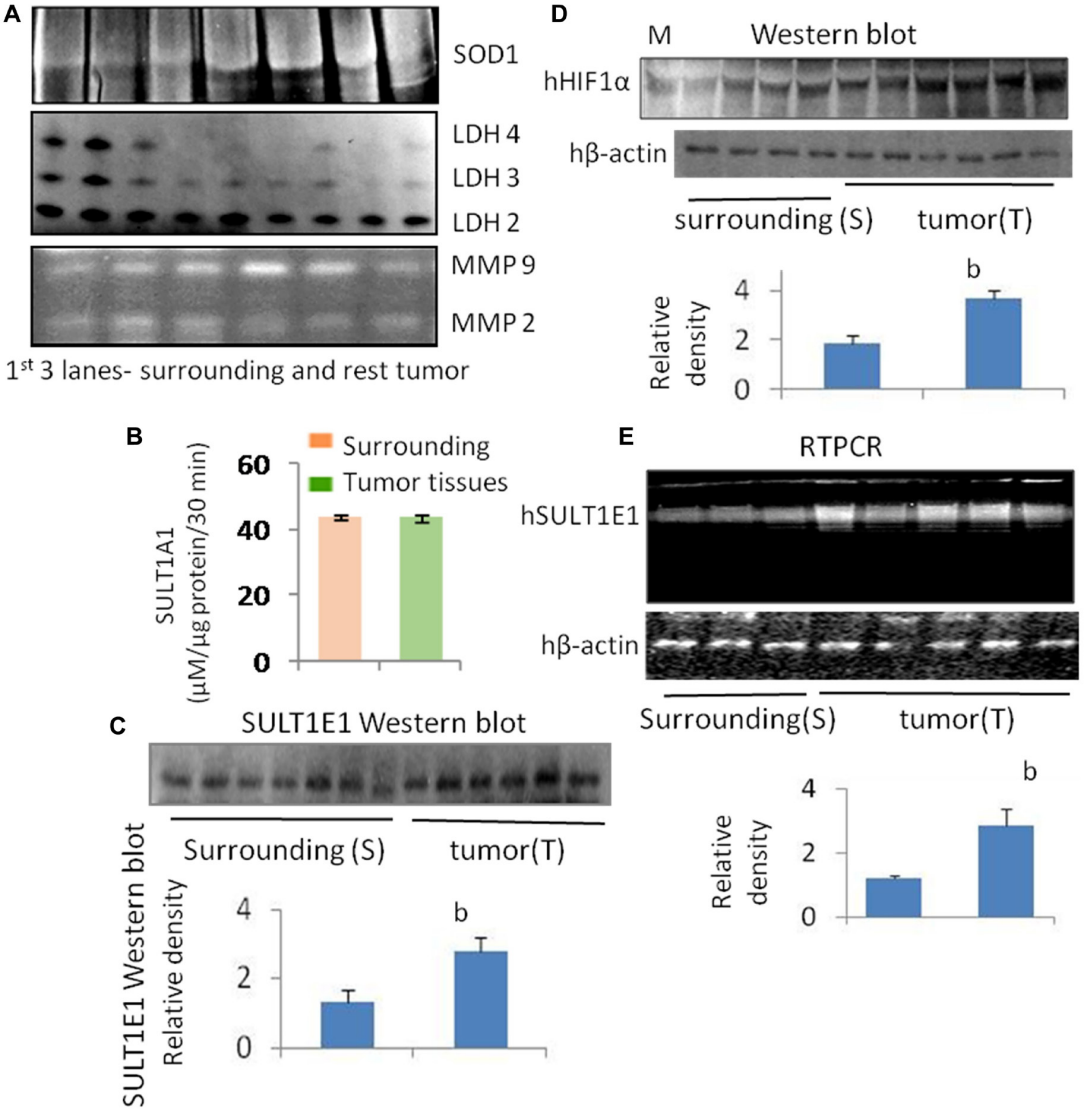
(**A**) Expression of SOD1, LDH 2, 3 and 4, and MMP2, MMP9 in human breast tumor (*n* = 3) and its corresponding surrounding tissues (*n* = 3): Lane distribution-1, 2, 3-surounding and 4, 5, 6 –Tumor. (**B**) Activity assay of SULT1A1 in human tumor tissue (*n* = 3) and its corresponding surrounding areas (*n* = 3). (**C**) SULT1E1 (protein) expression in tumor (*n* = 6) tissue and their corresponding surrounding (*n* = 6) tissue are calculated and densitometric data are presented. (blot image not shown). Level of significance in Student’s *t*-test is, b = *p* < 0.01. (**D**) HIF1α (protein) expression in tumor tissue (*n* = 6) and their corresponding surrounding (*n* = 4) tissue with densitometric analysis. Molecular weight marker β-galactosidase (120 kD) was used in this study. Human β-actin was used to verify the control protein. Level of significance in Student’s *t*-test is, b = *p* < 0.01. (**E**) SULT1E1 (gene) expression RTPCR data in tumor (*n* = 5) tissue and their corresponding surrounding (*n* = 3) tissue are calculated and densitometric data are presented. Level of significance in Student’s *t*-test is, *b* = *p* < 0.01.

The protein density and mRNA density (in surrounding and tumor) from western blot and RT-PCR data suggests that both protein density (PD) and mRNA density (MD) of 1E1 and HIFα are significantly increased in the tumor groups (Table 1 and Figure 1). Present correlation study suggests that tissue MDA level is positively associated with SULT1E1 tissue expression. The 1E1 is an adaptive enzyme that catalyze the inactivation of E2 by forming E2S. But in breast cancer tissue it is noticed that, at higher disease state with prolonged oxidative stress exposure and high MDA level HIFα is increased significantly and is shown to positively associated with HIFα induction. NPSH and uric acids are regarded as the endogenous soluble antioxidants those may have some protective role against the oxidant-stress-induced 1E1 regulations, HIFα induction. Present results and correlation data suggest that NPSH and uric acids are negatively correlated with HIFα expression. Lower antioxidant level augments the HIFα level (Table 2). Table 3 suggests that in control, DAS and Chalcone groups, NPSH and 1E1 are found to be associated. When in control and in DAS these are positively associated in Chal- group NPSH and 1E1 are shown to be negatively associated. No significant association was noticed in DAS and Chalcone groups.

**Table 1:** The protein density and mRNA density (in surrounding and tumor) from western blot and RT-PCR data

Group statistics								
Type		N	Mean	SD	SE	*t*	One-sided *p*	Two-sided *p*
SULT_PD	Surr	6	1.6667	0.81650	0.33333	−3.138	0.005	0.011
	Tumor	6	2.9333	0.55737	0.22755			
HIFaPD	Surr	4	1.8750	0.85391	0.42696	−2.863	0.011	0.021
	Tumor	6	3.6667	1.03280	0.42164			
SULT1E1_mD	Surr	3	1.2000	0.20000	0.11547	−2.857	0.018	0.036
	Tumor	4	3.2250	1.18708	0.59354			

**Table 2:** Statistical analysis of Pearson’s correlations amongst different oxidative-stress parameters and SULT1E1 expressions in three different diseases conditions in the tumor/surrounding tissues

Correlations						
		MDA	NPSH	URIC_ACID	HIFa_Expr	SULT1E1_Expr
MDA	r	1	0.090	0.026	−.509^**^	.486^**^
	P		0.611	0.888	0.006	0.006
	N	34	34	32	28	30
NPSH	r	0.090	1	0.138	−.605^**^	0.159
	P	0.611		0.452	0.001	0.402
	N	34	34	32	28	30
URIC_ACID	r	0.026	0.138	1	−0.364	0.282
	P	0.888	0.452		0.068	0.145
	N	32	32	32	26	28
HIFa_Expr	r	−.509^**^	−.605^**^	−0.364	1	−.518^**^
	P	0.006	0.001	0.068		0.005
	N	28	28	26	28	28
SULT1E1_Expr	r	.486^**^	0.159	0.282	−.518^**^	1
	P	0.006	0.402	0.145	0.005	
	N	30	30	28	28	30

^**^Correlation is significant at the 0.01 level (2-tailed).

**Table 3:** The correlation data suggest that in control, DAS and chalcone groups, NPSH and SULT1E1 are found to be associated

Correlations				
Type			NPSH	SULT1E1
CONTROL	NPSH	Pearson Correlation	1	.944^**^
		Sig. (2-tailed)		0.005
		N	6	6
	SULT1E1	Pearson Correlation	.944^**^	1
		Sig. (2-tailed)	0.005	
		N	6	6
DAS	NPSH	Pearson Correlation	1	.931^**^
		Sig. (2-tailed)		0.007
		N	6	6
	SULT1E1	Pearson Correlation	.931^**^	1
		Sig. (2-tailed)	0.007	
		N	6	6
CHAL	NPSH	Pearson Correlation	1	−.855^*^
		Sig. (2-tailed)		0.030
		N	6	6
	SULT1E1	Pearson Correlation	−.855^*^	1
		Sig. (2-tailed)	0.030	
		N	6	6
CHAL,DAS	NPSH	Pearson Correlation	1	0.159
		Sig. (2-tailed)		0.763
		N	6	6
	SULT1E1	Pearson Correlation	0.159	1
		Sig. (2-tailed)	0.763	
		N	6	6

^**^Correlation is significant at the 0.01 level (2-tailed). ^*^Correlation is significant at the 0.05 level (2-tailed).

Table 4 suggests that, in case of time-based exposure NPSH and 1E1 and 1E1 are found to be positively associated and 3 and 24 hrs. of exposure. No significant relation was noticed in 5 hrs. exposure. New sample size and internal variability may be the cause for this.

**Table 4:** Time dependent correlation data of different groups of DAS, chalcone exposed rat

Correlations				
Hour			NPSH	SULT1E1
3.00	NPSH	Pearson Correlation	1	.895^**^
		Sig. (2-tailed)		0.003
		N	8	8
	SULT1E1	Pearson Correlation	.895^**^	1
		Sig. (2-tailed)	0.003	
		N	8	8
5.00	NPSH	Pearson Correlation	1	0.606
		Sig. (2-tailed)		0.111
		N	8	8
	SULT1E1	Pearson Correlation	0.606	1
		Sig. (2-tailed)	0.111	
		N	8	8
24.00	NPSH	Pearson Correlation	1	.828^*^
		Sig. (2-tailed)		0.011
		N	8	8
	SULT1E1	Pearson Correlation	.828^*^	1
		Sig. (2-tailed)	0.011	
		N	8	8

^**^Correlation is significant at the 0.01 level (2-tailed). ^*^Correlation is significant at the 0.05 level (2-tailed).

### SULT1E1 protein expression in tumor and surrounding tissues

Expression of SULT1E1 protein in tumor was comparatively higher than corresponding surrounding tissues. A few surrounding tissues have shown an increased SULT1E1 expression, whose corresponding tumor shows more intense SULT1E1 expression. Our findings abide by earlier study that suggests that SULT1E1 is expressed more in the tumor tissues as compared to their surroundings, and our study is the first attempt to explain that, though SULT1E1 is overexpressed in tumor it remains inactive [20].

### HIF1α protein expression in rat liver tissue treated with DAS and chalcone

The expression of HIF1α in tumor tissue was remarkably higher when compared to their corresponding surrounding tissues which was noticed in SULT1E1 expression. HIF-1 plays important roles in breast cancer metastasis by mediating hypoxia-induced expression of mRNA-encoding genes. HIF-1 also regulates the expression of non-coding RNAs, which are critical regulators of migration, invasion, and metastasis [14].

### Superoxide dismutase (SOD1) and Lactate dehydrogenase (LDH) activities in breast cancer tissue

The antioxidant enzyme superoxide dismutase (SOD) activity was found to be higher in the tumor as compared to its surrounding (Figure 1A, SOD1). In essence, lactate dehydrogenase (LDH) is an enzyme necessary for the conversion of sugar into cellular energy. An elevated total serum SOD level is a predictor of tissue damage and inflammation [21]. Another study has shown loss of LDH-B expression as an early and frequent event in human breast cancer [22]. In our current study, we found the loss of LDH 3, 4 and 5 in breast cancer as compared to the surrounding whereas LDH 2 seems to be equally expressed both in the surrounding and the tumor. LDH has 2 subunits, subunit A and subunit B in different proportion in each type of LDH. In this study, we found that the expression of subunit A is reduced or almost absent in breast tumor. Therefore, the less is the expression of LDH 3, 4 and 5 in tumor tissue (Figure 1A).

### Matrix metalloproteases (MMP 2 and 9) activities

Breast tissue expressed both MMP2 and MMP9. Compared to the surrounding tissue, the tumor tissue had a higher expression of MMP9 (Figure 1A). MMP9 was not similarly expressed in all the tumors, some tumors had less, whereas some had more MMP9. MMP2 expression was less in the tumor in comparison to the corresponding surroundings. MMPs are vital in cancers. MMP-9 can cleave many extracellular matrix (ECM) proteins to regulate ECM remodeling. Several plasma surface proteins can also be cleaved by it, releasing them from the cell surface. MMP-9 has been widely found to relate to the pathology of cancers, including but not limited to invasion, metastasis and angiogenesis [23].

### SULT1A1 activity of human breast cancer sample

Sulfotransferase also utilizes PAPS as sulfonate donor to catalyze the sulfate conjugation of a wide variety of acceptor molecules bearing a hydroxyl or an amine group including estrogen. To find whether E2 is controlled by other E2 regulating enzymes such as SULT1A1, we did SULT1A1 activity assay. Interestingly we found no difference in the SULT1A1 activity between tumor and their surrounding tissue (Figure 1B). Densitometric data are displayed along with the calculation of SULT1E1 (protein) expression in tumor (*n* = 6) tissue and the comparable surrounding (*n* = 6) tissue. (No image of blot provided). The Student’s *t*-test has a significance level of b = *p* < 0.01 (Figure 1C). Densitometric examination of the expression of the protein HIF1α in six tumor samples and the corresponding four adjacent tissues. In this investigation, the molecular weight marker β-galactosidase (120 kD) was employed. The control protein was validated using human β-actin. The Student’s *t*-test has a significance level of b = *p* < 0.01 (Figure 1D). Densitometric data is displayed along with the SULT1E1 (gene) expression RTPCR data in tumor (*n* = 5) and adjacent (*n* = 3) tissue. The Student’s *t*-test has a significance level of b = *p* < 0.01 (Figure 1E). Thus, there is no SULT1A1 interference in the final outcome of E2.

### Histopathology of human breast cancer sample

**Figure 2:**
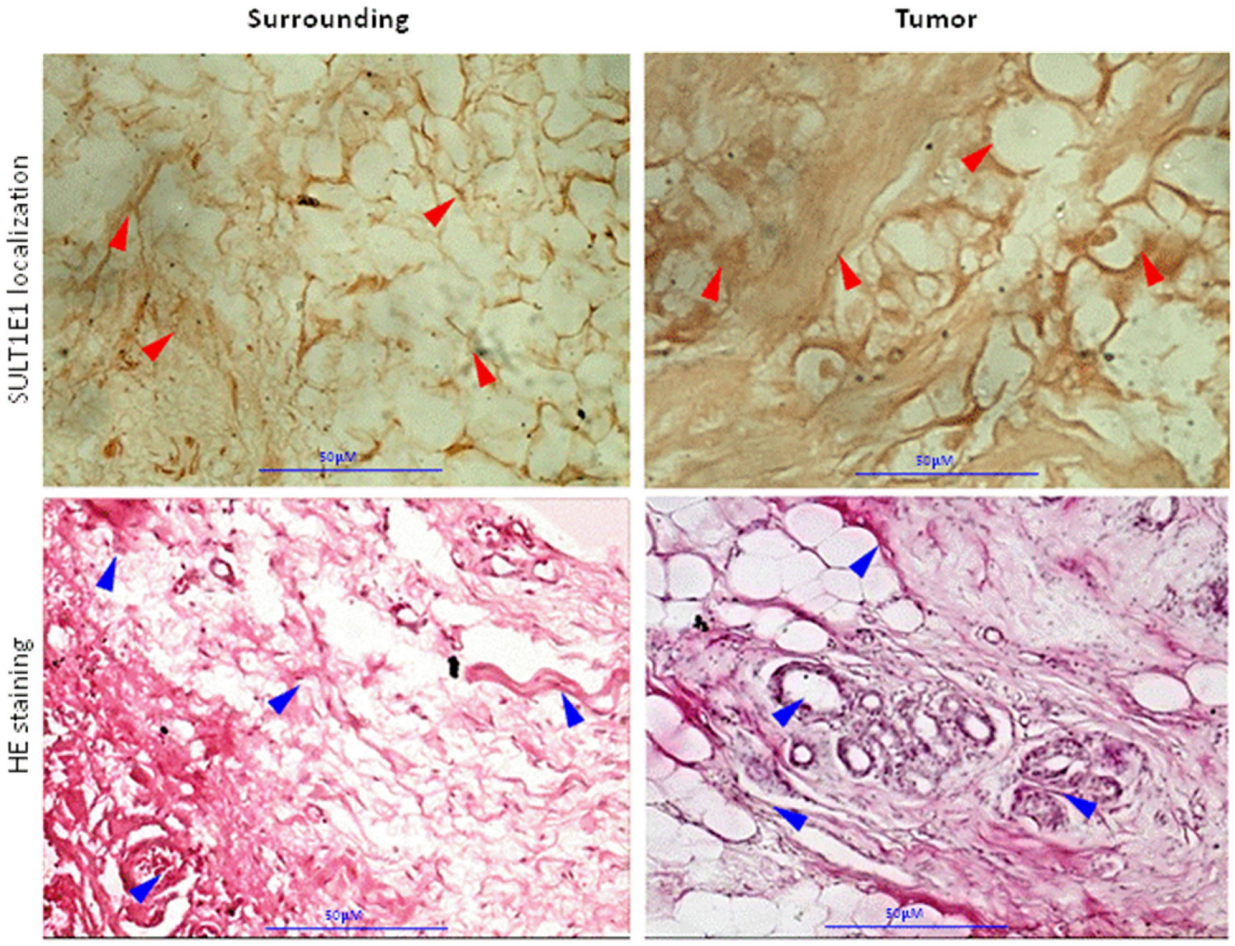
Immunohistochemistry- SULT1E1 expression in tumor tissue and adjacent surrounding tissue. HE staining- Histoarchitecture of tumor and adjacent surrounding tissue.

In the tissue surrounding the tumors, few normal ducts lobules in a fibrous stromal along with adipose tissue are identified (Figure 2, Surrounding). The tumor tissue shows ductal carcinoma *in-situ* and micro invasive cells. Hematoxylin-eosin stained breast cancer tissue shows several mitosis and pleomorphic nuclei. Irregular and undifferentiated ducts are present in the tumor tissue (Figure 2).

### Immunohistochemistry of SULT1E1 of human breast cancer sample

Immunohistochemistry results show that the tumor and surrounding both are positive for SUT1E1. The strength of SULT1E1 staining in tumor is stronger than corresponding surrounding tissue. The distribution and localization of SULT1E1 was noticed in stromal tissue, adipose lining and nucleus in both tumor and corresponding surrounding tissues. Tumor tissue was darkly stained for SULT1E1 compared to the surrounding suggesting increased expression (Figure 2).

### Catalase and SOD activities after exposure of drugs for varied durations

**Figure 3:**
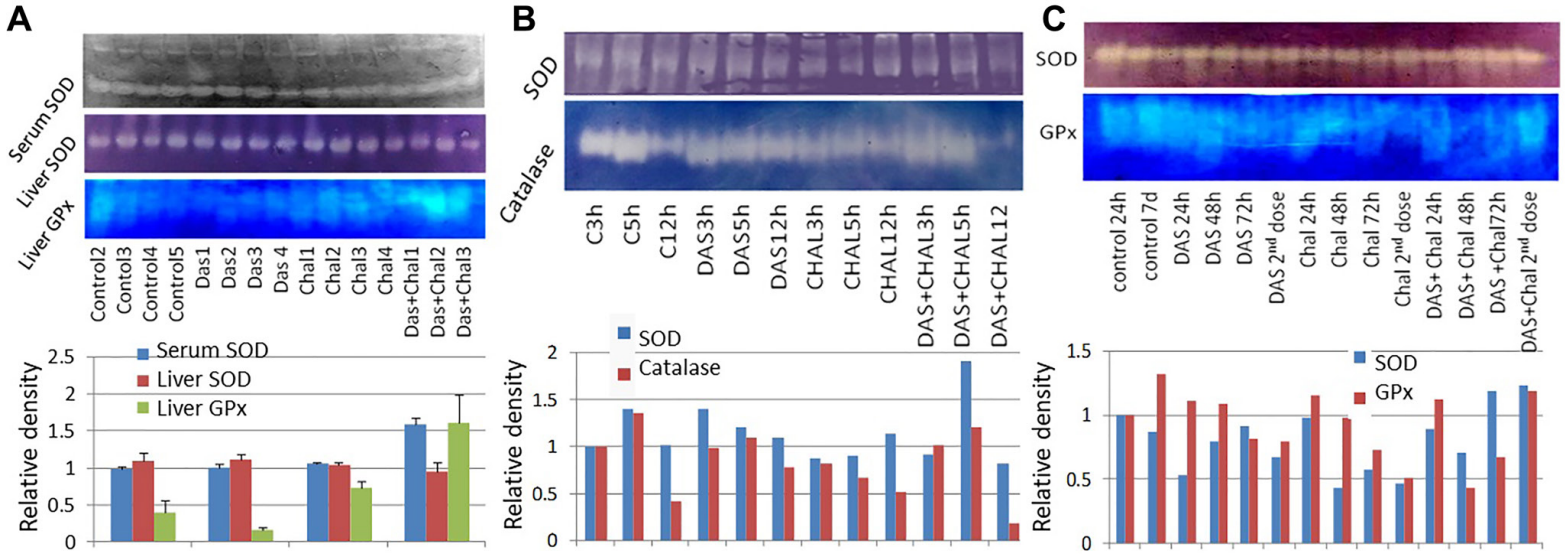
(**A**) Gel zymographic data of SOD activity in serum and liver tissue and GPx activities in liver tissues of rats treated with DAS, chalcone, or DAS + chalcone. (**B**) Gel zymographic data of SOD and catalase activity in the liver tissues of rat treated for 3, 5 or 12 hours with DAS, chalcone or DAS + chalcone. (**C**) Gel zymographic data of SOD and GPx activity in liver tissue of rats treated for 24, 48 and 72 hours with DAS, chalcone or DAS + chalcone. Relative densitometry data have been presented as the bar diagram at the bottom panel of gel-band figure.

The SOD activity was slightly less in the DAS group as compared to the control. The chalcone group also displayed lower SOD activity in half of the animal as compared to the control and DAS group. The other half shows SOD activity similar to the control group. The entire group showed comparatively lower SOD activity compared to the control (Figure 3A).

The enzymatic activity of catalase was low in DAS, Chalcone and DAS + Chalcone combination group in comparison to the untreated tissue. Catalase Activity gradually decreased in the in the DAS treated tissues with time, and the same was true for the Chalcone treated tissue. Among all the treated groups the highest activity was found in the DAS + Chalcone treated tissue for 3 and 5 hours, however no activity was found in the DAS + Chalcone- 24 hrs. treated tissue (Figure 3B).

The control group showed SOD activity, and DAS at 3 hour showed SOD activity but 5 and 12 hours showed negligible SOD. DAS + Chalcone at 5 and 12 hours showed some activity but less than the control (Figure 3B).

The SOD activity remained unchanged in the DAS alone, chalcone alone group with a slight decrease of activity in DAS group. The combination group showed a decrease in the activity in the DAS + Chalcone (48 hrs,) and then markedly increased in the 72 hrs. group and the group treated with the 2nd dose. DAS alone and Chalcone alone group showed comparatively less SOD activity than the control or untreated group. The combination group of DAS + chalcone showed a significant increase in SOD activity on treatment with the 2nd dose after 72 hours of the 1st dose. The chalcone treated group had low SOD activity than control and DAS group, but the 2nd dose had high SOD activity. Interestingly DAS + Chal had the highest SOD activity, which gradually increased with duration till 72 hrs. (Figure 3C).

### Glutathione peroxidase (GPx) activity study by gel zymogram

The GPx activity was highest in the control group (untreated) as compared to another group of animals such as DAS alone, Chalcone alone and DAS + Chalcone group. The GPx activity was significantly lower in the DAS group than it was in the chalcone, which was likewise significantly lower. The DAS + Chalcone group a gradual increase in the GPX activity with duration and the highest activity among them all was found in the DAS + Chalcone 2nd dose group (Figure 3C).

The control group showed some GPx activity and there was no activity in the DAS treated animal group. The Chalcone group showed minute activity. The Das + Chal group showed high GPx activity. The GPx activity was found to be lower in the DAS, Chalcone group as compared to the control group of animals. The GPx activity was stimulated in the DAS + Chalcone group of animal’s hepatic tissue (Figure 3A).

### SULT1E1 protein expression (animal experiment)

#### *In vivo* experiment

**Figure 4:**
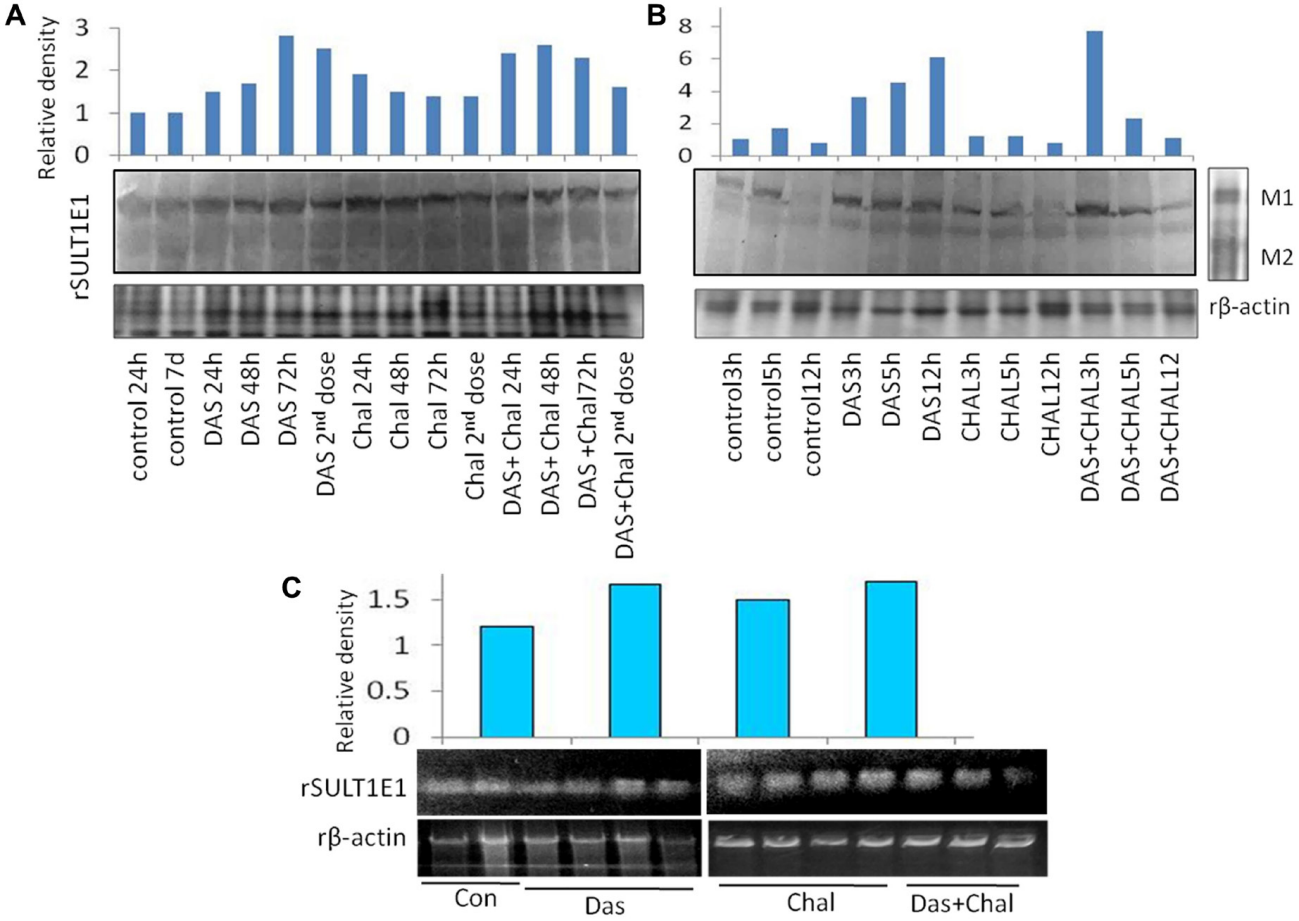
(**A**) Expression of rSULT1E1 protein and its densitometric analysis from rat treated with DAS, chalcone (Chal) or DAS + chalcone for 24, 48 and 72 hours and in one group DAS + chalcone with a second dose of DAS. Expression of rβ-actin was studied from all these experimental groups. Lane distribution- details are mentioned at the bottom of the lanes. (**B**) Expression of rSULT1E1 protein and its densitometric analysis in rat liver cells (primary culture) treated with DAS, chalcone or DAS + chalcone for 3, 5 and 12 hours. Lane distribution- details are mentioned bottom of the lanes. Two molecular weight marker M1 and M2 were also run to verify SULT1E1 protein. (**C**) Expression of rSULT1E1 mRNA from the liver of rat treated with DAS, chalcone or Das + chalcone and their densitometry data are presented. Expression of rβ-actin mRNA was also screened as a loading control.

**Figure 5:**
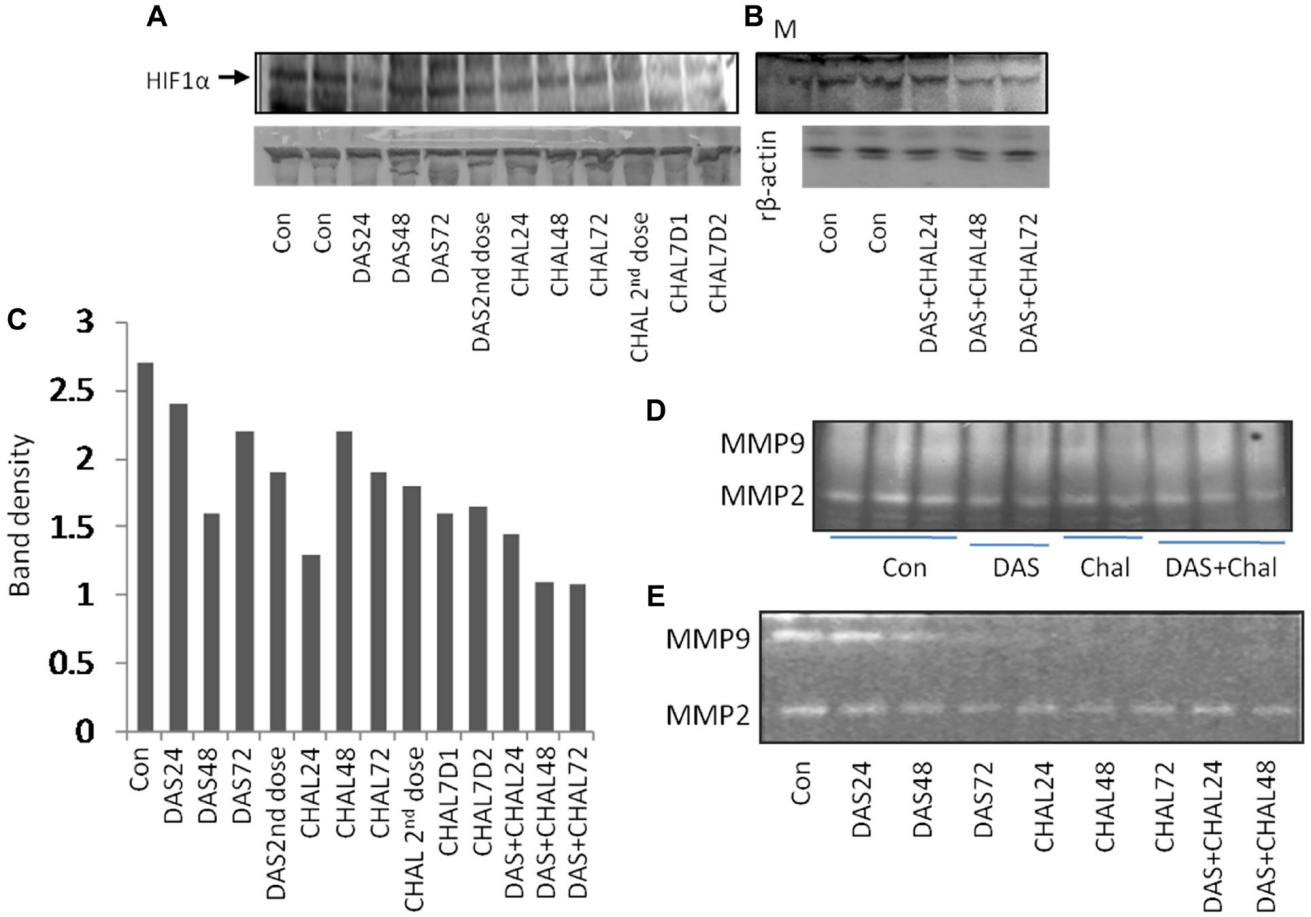
Expressions are demonstrated of rHIF1α protein in rat model treated with DAS, chalcone (Chal) for different time periods (**A**) and DAS + chalcone for 24, 48 or 72 hours (**B**). Densitometric analysis and relative band densities of (A, B) are presented as bar in the diagram (**C**). Gel zymographic data of MMP2 and MMP9 from the liver tissues of rat treated with DAS, chalcone or DAS + Chalcone for 7 days treatment (**D**). Gel zymographic data are shown of MMP2 and MMP9 from the rat liver tissues treated *in vitro* with DAS, chalcone or DAS + chalcone for 24, 48 or 72 hours (**E**).

**Figure 6:**
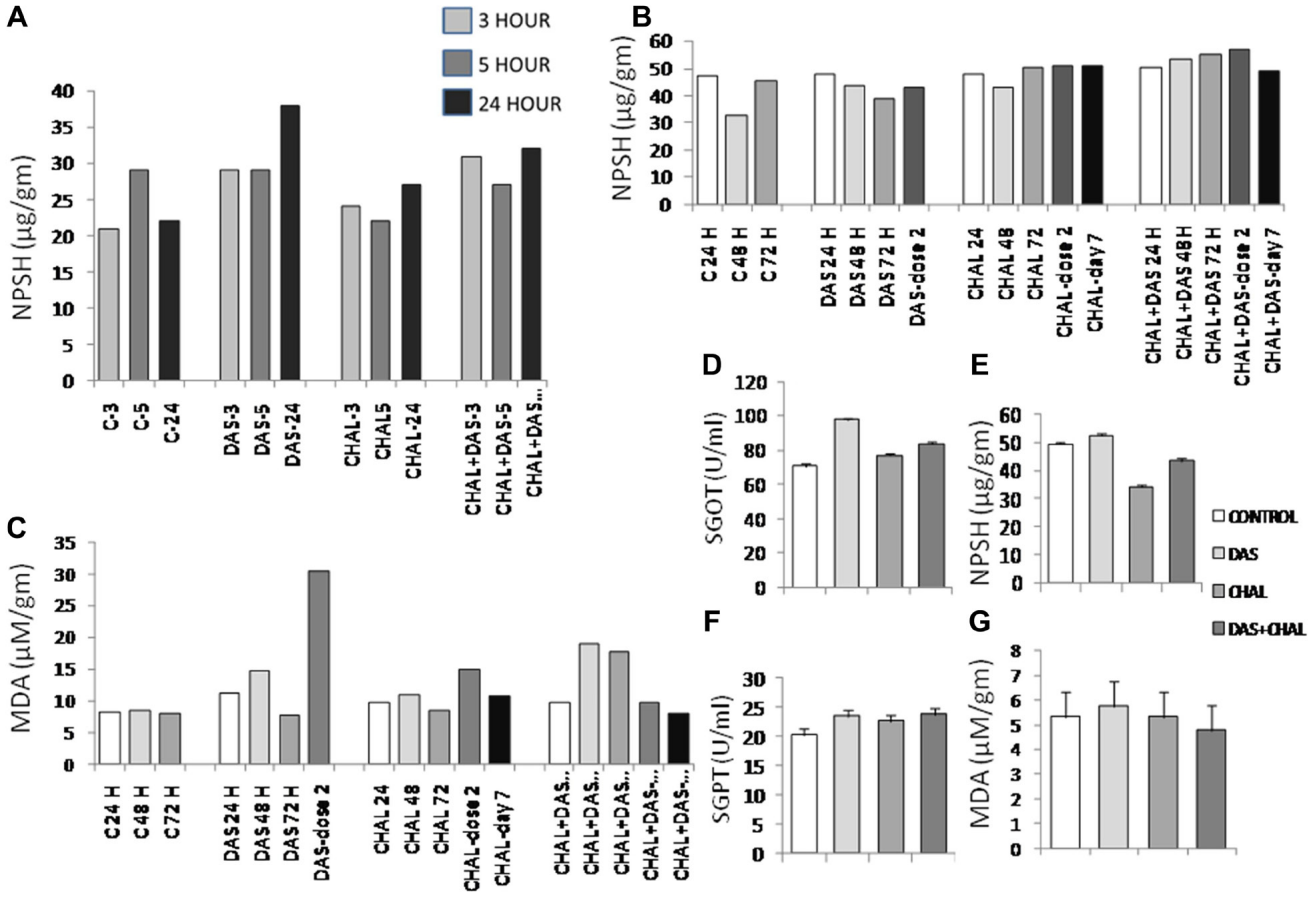
Different parameters were measured from rat liver tissue after *in-vitro* treatment with DAS, chalcone or DAS + chalcone and data are presented as bar diagram. The representation of bars is mentioned in the figures-Individual data from *in vitro* experiments are presented in (**A**–**C**). Different groups had 3–5 animals per group as depicted in the picture and animals were serially sacrificed at stipulated time interval. *In vivo* experimental data from 7 days treatment groups are presented in (**D**–**G**). Bars represent the means ± SE from 4 animals in each group. Levels of significances of difference between two groups are verified Student’s *t*-test.

Following a single dosage of DAS on day 1, the hepatic tissue animal group treated with DAS demonstrated a progressive increase in the SULT1E1 expression with increasing duration. SULT1E1 expression was comparable to DAS 48 hours after DAS 2nd dose group. On the other hand, in 24- and 48-hours groups of chalcone treated animals expressed highest SULT1E1 but the expression gradually decreased with increasing duration. The combination group (DAS + Chal) showed an expression level less than the DAS or Chalcone alone group but greater than the control group (Figure 4A). Present results and correlation data suggest that NPSH and uric acids are negatively correlated with HIFα expression. Lower antioxidant level augments the HIFα level (Table 2). The data from this table are represented from DAS and chalcone related studies shown in the Figures 4–6.

#### *In vitro* experiment

The DAS group expressed SULT1E1 more than the control group during the *in-vitro* therapy, and this expression gradually increased over time. The chalcone group had less SUT1E1 expression than the control and gradually decreased with increasing time. Whereas the combination group had highest expression at the first 3 hrs. of incubation, decreased at 5 hours and remain undetected at 12 hours of incubation (Figure 4B). However, 3 hrs DAS + Chal treated tissue showed the best result with highest level of SULT1E1 and then gradually decreased in 5 hrs. and least is found in 12 hrs. Thus, DAS has direct effect on the expression of SULT1E1 and Chalcone has some moderate effect.

### Messenger RNA (mRNA) expression studies of SULT1E1

When administered for a period of 7 days, it was discovered that DAS and Chalcone, both alone and collectively, were able to increase mRNA in comparison to the animals in the control group via inducing transcription of the SULT1E1 gene. SULT1E1 mRNA was highly expressed in the DAS treated cells. Chalcone also expresses SULT1E1 mRNA but less than DAS. Whereas DAS + Chal treated showed highest SULT1E1 in comparison to DAS alone and Chalcone alone group but a single animal had low SULT1E1 mRNA. These expressions were all higher than the control group (Figure 4C).

### Expression of HIF1α protein

HIF1 expression was high in the DAS-24 hrs. group and then appeared to gradually decline with time as well as with the second dosage. Yet, in the Chalcone-treated group, HIF1 is observed to gradually express more with time and the second dose. In the DAS + Chalcone group of animal hepatic tissue, the expression of HIF1 was essentially the same. This justifies why DAS + Chal have little impact on the HIF1 group (Figure 4A, 4B). The band density of HIF1 expression is depicted in the bar diagram, which also clearly shows the pattern of HIF1 expression. (Figure 4C).

In a rat model administered with DAS, chalcone (Chal) for varying durations the rHIF1α protein are presented (Figure 5A) and the same protein expression are demonstrated in DAS + chalcone for 24, 48, or 72 hours groups (Figure 5B). In both pictures it is clear that DAS and chalcone alone or in combination decreased HIF1α. The graphic data (Figure 5C) displays the relative band densities as the protective role of these drugs and densitometric analysis of Figure 5A, 5B. The MMP2 and MMP9 gel zymographic data are demonstrated from rat liver tissues treated with DAS, chalcone, or DAS + chalcone for seven days (Figure 5D). MMP2 and MMP9 gel zymographic data from rat liver tissues treated *in vitro* for 24, 48, or 72 hours with DAS, chalcone, or DAS + chalcone are displayed in Figure 5E. These data suggest that DAS and chalcone could be able to inhibit MMPs action.

### Oxidative stress related parameters; malondialdehyde (MDA) and non-protein soluble thiol (NPSH)

The level of non-protein thiol *in vitro* experiment increased after 3, 5 and 24 hrs. of incubation with DAS. Das + Chalcone incubation also causes NPSH induction as compared to the control cells (Figure 6A). In the *in vivo* experiment, NPSH was negligibly increased in the DAS alone and Chalcone alone treated group, whereas a slight increase was noticed in DAS + Chal, 48 hrs, 72 hrs. and 2nd dose treatment (Figure 6B). Serum NPSH was found to be reduced in the Chalcone alone group, and the other entire group had NPSH similar to the control, showing no changes in the redox status in terms of NPSH.

Elevated MDA was seen in DAS-treated mice, demonstrating high MDA production with the second dose of DAS, suggesting that DAS does appear to trigger lipid peroxidation. During 48 and 72 hours, Chalcone 2nd dosage and DAS + Chalcone also had a higher MDA, in contrast to the other groups, which displayed MDA similar to the control group (Figure 6C). Hence, these medications lacked such liver damage. So, these drugs can be used as therapeutic agents (Figure 6D). Serum MDA levels remain stable (Figure 6E). This means that neither DAS nor Chalcone cause systemic lipid peroxidation. Figure 6F demonstrates Serum Glutamate Pyruvate Transaminase (SGPT) activity in the drugs-treated rat hepatocytes. A slight increase in the enzymatic activity was noticed in these groups. Malondialdehyde (MDA) levels were demonstrated in Figure 6G which showed basically no significant changes in different groups.

## DISCUSSION

Our present result of DAS induction of SULT1E1 has been supported by a previous finding of 1E1 induction by this drug in mouse liver. Recently, mouse SULT1E1 was shown to be induced by many chemicals/compositions including garlic extract (*Allium sativum*, rich with DAS) that activate CAR [18]. This suggests that use of crude garlic extract may have some beneficial effect in E2 dependant cancer.

In this study, we sought to combine the idea of SULT1E1 induction by DAS with the creation of a reducing environment by chalcone. An enhanced level of SULT1E1 has been found in tumor tissues in earlier research as part of an adaptation mechanism to control active E2. However, the 1E1 activity may be insufficient to counter the significant surge of E2 in E2 dependent breast cancer. Limitations in 1E1 activity might be due to its inactivation in oxidative stress in breast cancer [20], where Cys83 of SULT1E1 may remain in oxidized state and hinders E2 binding and sulfo-conjugation by this enzyme [20]. Eventually, it allows elevated active estrogen leading to a carcinogenic effect. This study hypothesized that the induction of SULT1E1 by DAS and its activation by chalcone may significantly control E2 level and the disease pathogenesis resulting in an increase in patients’ survival. This abides by an earlier study that SULT1E1 is an oxidative stress responsive gene that gender specifically affects liver/reperfusion injury [20, 24]. Chalcone has been shown to create a reducing environment that helps to keep SULT1E1 active in the present study.

Both *in vivo* and *in vitro* experiments provide us a clear outlook that this drug combination may be an effective therapeutic strategy against E2 dependant breast cancer. Oxidative stress is known to induce HIF1α via Nrf2 which has been shown to be induced in human ovarian cancer may have possible role in breast cancer also. Role of Nrf2 and NfkB has been demonstrated in human breast cancer tissue and in experimental rodent model [20, 25]. A link between Nrf2 and HIF1α led us to investigate HIF1α, since Nrf2 is completely associated with oxidative stress management. Oxidative stress is linked with SULT1E1 induction and activation via Nrf2 [26].

Potential experimental models of rat hepatocytes exposed to current drugs were used to test their metabolism and their ability to regulate hepatic gene expressions [27, 28]. In the current study, beside its reducing effects, chalcone showed strong antioxidant effect and activate the catalase and both drugs strongly activate the SOD activity also. Superoxide dismutase and catalase mimetic-drugs MnTmPyP and 134 have distinct impacts on breast cancer cell proliferation via TNFa-induced NF-B regulations confirm our findings that proposed drugs may have positive effects [29]. This result may be linked to our previous study on human breast tumor sample [20]. Anti-invasive and anti-angiogenic potential of chalcone derivatives acted as an HIF-1 inhibitor [30] which is also noticed in our study.

DAS and chalcone mediated induction of anti-stress responses may be via activated Nrf2, thereby, increasing of antioxidant enzyme i.e., SOD, catalase and GPx supporting to oxidative-stress induction and its possible adaptations have been noticed in the current study. The novel combination of DAS and chalcone might be invaluable, because oxidative stress not only inactivates SULT1E1 but also augments Nrf-2 and HIF-1a in breast cancer. This ascertains the induction of the reducing environment and minimization of oxidative stress which might be strongly supportive in E2 related cancers. However, there is some amount of lipid peroxidation products (MDA) are noticed in the liver tissue of the rats of 2nd dose DAS and DAS + Chal groups (48 and 72 hours). An extra amount of antioxidant (vitamin C and E) as supportive to this combination would be able to protect the tissue. Report suggests that some chemotherapeutic applications may cause some level of systemic stress. As a result of decreased glucose absorption and the production of lactic acid, it significantly aided ROS-induced apoptosis in breast cancer cells [31].

Cancer subclones are encouraged by intramural hypoxia in metastasis. HIF-1 expression levels at various clinical phases of the illness predict the outcome for several malignancies, including breast cancer. Tumor tissue in the current study has higher HIF1 protein expression than the surrounding tissue. DAS and Chalcone had a substantial inhibitory effect on the HIF1. This finding confirms our hypothesis that the use of these medications in combination provides a high level of clinical benefit. Our current study was supported by a previous study which has shown that diallyl trisulfide (DAS) dose-dependently inhibited HIF-1α in hypoxic MDA-MB-231 cells thereby inhibiting hypoxia-induced pre metastatic changes and angiogenesis in these cells. The angiogenic responses in high rate of tumorigenic growth may be terminated by the combined effects of DAS and Chalcone in addition to their SULT1E1induction effects.

**Figure 7:**
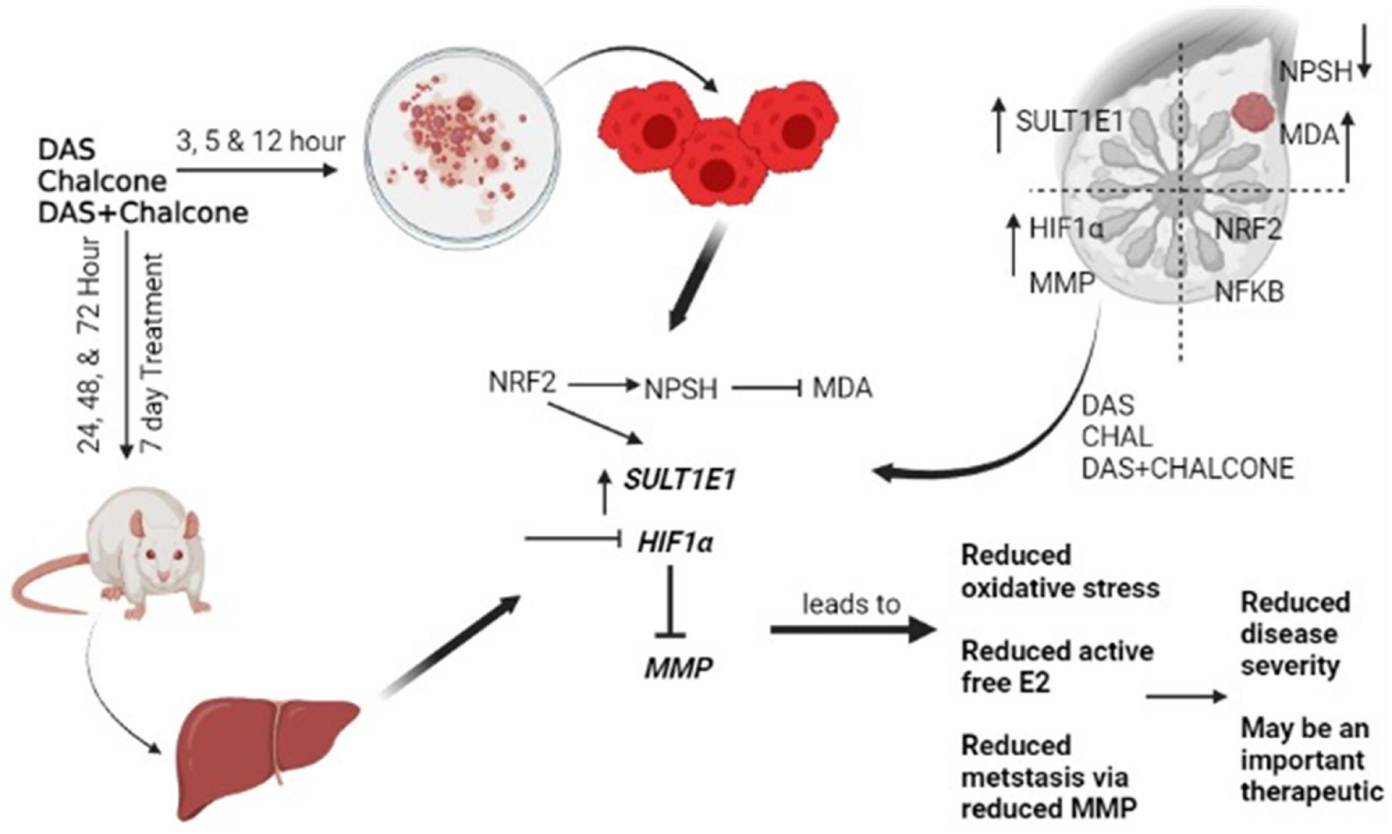
Schematic representation of breast cancer association with redox-regulated SULT1E1 dysfunction and HIF1a/MMPs up-regulations. The prominent therapeutic role of Dialyl-sulfide (DAS) and chalcone via reversal of these protein dysfunctions has been summarized.

The MMP9 was mainly expressed in tumor tissue and less expressed in surrounding tissue; some of those have higher and variable expressions due to inter-individual variability. The MMP2 was expressed less in the tumor tissues. The MMP has been widely found to relate to the pathology of cancers including but not limited to invasion, metastasis and angiogenesis [23]. Under hypoxic stress, HIF-1a rapidly accumulates and activates hundreds of genes including MMPs in breast cancer patients (Figure 7) [32]. Our preliminary findings on the potential control of breast cancer and metastatic status by using DAS and chalcone combination may be significant from a therapeutic standpoint. Chalcone also triggered the mitochondrial apoptotic signaling by increasing the amount of Bax and Bak and reducing the level of Bcl-2 and Bcl-X (L), and subsequently activated caspase-9 in MCF-7 and MDA-MB-231 cells [33].

Lactate dehydrogenase is responsible for conversion of sugar into energy in cells. Alterations in LDH may be related to anaerobic oxidation and metabolic status that eventually occur in hypoxic tissues that have been damaged by cancer. Earlier studies show loss of LDH-B expression as an early event that is frequently occurs in breast cancer [22]. We found loss of LDH 3, 4 and 5 in breast cancer in comparison to the surrounding and LDH 2 seems to be equally expressed both in the surrounding and the tumor. In the current study expression of Subunit A is reduced or almost lacking in breast tumor. Our findings interpret that, less is the expression of LDH 3, 4 and 5 in tumor tissue Once again this is also related to the HIF1a responses noticed in the current study [34]. Report showed a higher LDH-A expression in the tumor that has been associated to metastatic breast cancer. Supportive to our present LDH result I link may be pointed between hypoxia induced LDH and HIF1a expressions as different stages of breast cancer predictor [35].

The current result suggests that 1A1 mediated non-specific E2-sulafation is not taking place which supports more important role of 1E1. Altered regulations of 1A1 and/or its polymorphism have been linked to several types of cancer. However, under a chemotherapeutic setting the complex role of 1A1 cannot be easily predicted. As for example, possible interaction of anti-cancer anti-estrogenic drug tamoxifen with the SULT1A1 may be considered here. The anti-cancer therapeutic effect of tamoxifen and melatonin may be somewhat influenced by 1A1’s biotransformation of these medicines [36].

Present experimental drugs induced/activated several genes and proteins in this study. In addition, DAS might become a multifunctional drug that inhibits HIF-1α and induces SULT1E1at the transcriptional and translational levels. Previous studies from our lab have shown that SULT1E1 expression either increases in the tumor with increased oxidative stress at late stages of the disease or reduces at the initial stages of the disease to let E2 be active and gradually increases with the disease pathogenicity [20], but remains inactive due to elevated oxidative stress, as shown in our animal studies (ENU paper) and another earlier studies. The current study shows no alterations in the SULT1A1 activity in the breast tumor and surrounding tissue explaining that the breast cancer may have little or no role of SULT1A1 mediated nonspecific metabolism of estrogen. It is known that the liver is the port of entry and metabolism of any exogenous and endogenous drugs including estrogen which are significantly catabolized by the phase I and phase II enzymes. Liver produces and circulates (to target organs like breast, endometrium) significant amounts of estrogens and liver generates different estrogen-metabolizing enzymes like SULT1E1, STS and others. The mRNA and protein tested in the current investigation are highly expressed in liver at basal level and significantly respond by a modulator. It is shown that dexamethasone (DEX) treatment increased hepatic and MCF-7/VEGF tumor expression of *Sult1e1/SULT1E1* [35, 37]. Glucocorticoid receptor expression induced by DEX can also augment SULT1E1 expression in mouse liver and MCF-7 resulting in more E2 sulfation/inactivation and tumor growth inhibition [16, 38]. Liver is the competent source for study a large number of genes/proteins expression. Other important point is that liver is an important target of breast cancer metastasis. Report reveals that breast cancer liver metastasis (BCLM) is linked with poor prognoses of this disease. Tumor intrinsic subtype demonstrates preferential metastasis to liver with several types of breast cancer [39]. In this cancer, it is an adaptive strategy by increasing SULT1E1 gene and protein expression, tissues make an initial attempt to get rid of extra amount of E2. But due to more oxidative environment, SULT1E1 cannot perform properly. In the interim, as our study and several previous investigations have demonstrated, there is a high rate of cell division, particularly in the tumor region when compared to its surroundings. Oxidative stress also increases during this time, and a hypoxic environment exacerbates the situation by elevating the expressions of HIF1a, Nrf2, NfkB, and ultimately tissue-degenerating MMPs. So, to counteract the fast rate kinetics of tumor growth and adversely functioning of these genes/proteins, application of some therapeutic measure might help. In the current study DAS and chalcone combination has been shown to significantly increase SULT1E1 expression/activation, and decrease oxidative stress, HIF1a, MMPs expressions.

## MATERIALS AND METHODS

### Ethical clearance and fulfillments of other regulatory affairs

This is to state that the present study was carried out in accordance with the National Institutes of Health, USA guidelines and the institutional ethical concerns, relevant guidelines and regulations were maintained throughout the investigation. This study confirms that all experimental protocols were approved by the institutional Ethics Committee (oist/EC/hu/bt/16/). This is also to state that informed consent was obtained from all participants who were at their post-menopausal age.

Female Wistar rats were purchased from a small-animal firm house (Govt. registered) that follows all ethical norms and maintain requisite regulatory affairs. The firm house is a government accredited (CPCSEA-Committee for the Purpose of Control and Supervision of Experiments on Animals: Reg. No. 1A2A/PO/BT/S/15/CPCSEA. organization under the Dept of Animal Husbandry and Dairy, Ministry of Agriculture and Farmer’s Welfare, Govt. of India. Proper permissions for all animal experiments, were obtained from the Institutional (Oriental Institute of Science and Technology) Review Board.

### Inclusion and exclusion criteria

Inclusion criteria- Patients only with breast carcinoma were included. Tumors were collected only from patients undergoing mastectomy. Those patients were also included who had a large gap of time between chemotherapy and mastectomy.

Exclusion criteria- Women suffering from endometriosis, pelvic inflammatory disease, tuberculosis, or any kind of liver and kidney disease, ovarian cancers, polycystic ovarian syndrome (PCOS), colon carcinoma, lung carcinoma, pregnancy, menstruation or any other infectious disease like HIV, HPV, HCV and Hepatitis B were excluded.

### Details of the participants

Detailed information about randomly selected patients (maintaining both-side anonimity and double-blind random screen) who donated their tumor samples has been provided in Supplementary Table 1. The Supplementary Table 1 informs about the patient’s age (44.69 ± 7.55 years; mean ± SD) and body weight (56.08 ± 6.63 Kg; mean ± SD), livelihood status, nutritional status, grade of the disease, tumor size and the details regarding the inclusion of the lymph nodes and metastasis.

### Breast tumor sample collection

The study was conducted in Oriental Institute of Science and technology and a total of 23 breast tumor samples were obtained from the local District Medical College and Hospital with proper ethical clearance. Breast tumors are diagnosed clinically; breast cancers were classified on the basis of TNM (The extent of the tumor (T), the extent of spread to the lymph nodes (N), and the presence of metastasis (M)) staging and grades. In some cases, down staging of cancer with chemotherapy was done prior to surgery and samples were collected. In this regard, this is to mention that tumor sample and corresponding surrounding tissues were collected separately soon after surgery and stored at −20°C. A small part of the tissue was also stored in formalin for histology and immunohistochemistry. We sincerely thank Dr. Guangping Chen of Department of Physiological Sciences of Oklahoma State University for providing the primary antibody against rSULT1E1 and hSULT1E1.

### Treatment of animal model (rats) with Diallyl sulfide and Chalcone

The animal experiments were designed to study short term, and long term effect of the drugs. The results may provide us with valuable information of duration required by these drugs to affect the desired expression and activation of SULT1E1. Three different sets of experiment were used for evaluating the effect of dialyl-sulfide and chalcone. Two of the experiment were *in-vivo* and one *in vitro* experiment.

### *In-vitro* (3 hour, 5 hour and overnight treatment)

#### Preparation of single cell suspension of rat hepatocyte

The animal experiments were performed in the institutional animal facility and surgery chamber. The fresh and laboratory acclimatized animals were anesthetized and then euthanized by intraperitoneal injection of pentobarbital sodium (50 mg/kg). Fifty grams of rat liver tissue processed to have single cell suspension. A nylon mesh was used to scrape the tissue in DBSS Buffer. The scarped product was centrifuged at 120 × *g*. The cellular pellet was washed with constituted L-15 media. A final suspension of the cells was made in 10 ml of constituted L-15 media.

Three *in vitro* groups based on duration of exposure were considered for this study. Grp 1–3 hr, Grp 2–5 hr. and Grp 3–12 hrs. exposure. Each petri dish was provided with 650 μl of cell suspension (from stock) and each group comprised of 4 plates containing a total of 10 ml solution such as Plate-1 (control): 650 μl of cell suspension + 9250 μl buffer, Plate-2: DAS-100 μM + buffer (making up to 10 ml), Plate-3: Chal-100 μM + buffer (making up to 10 ml), and Plate-4: DAS + Chal 100 μM each + buffer (making up to 10 ml).

### *In-vivo* (24, 48, 72 hours treatment)

Animals were divided in 3 groups, Group 1-control, Group 2-Diallysulfide (DAS), 80 mg DAS/100 gm b.w., Group 3- Chalcone, 4 mg/100 gm b.w., Group 4- Combination of DAS (80 mg) and Chalcone (4 mg). The animals were treated with the drug or drug combination by gavages. DAS and chalcone were solubilized in 0.1% ethanol (Ethanol water) to get the required concentration. The current dose of DAS and chalcone was screened after consultation with some previously published reports [17–19]. In addition, some dose response studies were performed in our lab to validate the selected dose. The present dose demonstrated significant responses on some gene’s expression like SULT1E1 and this dose did not initiate any toxicity which was evaluated from animal body weight, organ weight/body weight ratios, liver and kidney function test and hematology or other toxicity studies. Control group was given same amount of 0.1% ethanol solution as. These drugs were selected for animal exposure to test their ability to regulate SULT1E1 and HIF1α genes/proteins expressions. Different groups were assigned 3 animals per group anonymously in stock and those were serially sacrificed at 24, 48, 72 hours after drug administration, a second dose was given to a single animal at 72 hour and was sacrificed on the 5th day of the experiment i.e., at 120 hours or after 48 hours of the second dose. A single animal was sacrificed at the stipulated time point.

### *In vivo* (7-day treatment)

Four group having four animals each was taken for this experiment. Group 1 was control, Group 2 was treated per day with 20 mg of DAS, Group 3 was treated with 8 mg of chalcone and Group 4 was treated with a combination of DAS (20 mg) + Chalcone (8 mg). These animals were treated for 7 days and sacrificed on the 8th day. All animals were kept under UV radiation for 2 hours a day, until sacrificed. UV radiation was given for the purpose of immune compromization.

### Cytosol preparation of rat liver tissues

Immediately after animal sacrifice the liver tissue were cleaned in phosphate buffer and homogenized (30% w/v) in ice-cold phosphate buffer (0.1 mol /L, pH 7.4) then centrifuged at 10,000 rpm at 4ºC for 30 min. The supernatant (cytosol) was collected and stored at −20ºC in different aliquots for further assays.

### Cytosol preparation of human breast tissue sample

Breast tumor and the corresponding surrounding tissues were homogenized (30% w/v) in ice-cold phosphate buffer (0.1 mol /L, pH 7.4); and centrifuged at 10,000 rpm at 4ºC for 30 min. The supernatant (cytosol) was collected and stored at −20ºC in different aliquots for further assays.

### Western blot of HIF1α and SULT1E1

Western blot was conducted as in Maiti et al. 2007 with a slight modification. A 12% denaturing gel was loaded with 25 μg of protein (obtained from the rat liver tissue of animals treated with a single dose of DAS, Chalcone and DAS + Chal for 24, 48 and 72 hours) and electrophoresis carried out at 100v for 3 hours, transfer to nitrocellulose membrane was done at 100v for 2 hours. The membrane was washed and incubated with primary antibodies (anti rHIF1α and anti rSULT1E1) and secondary antibodies as mentioned in the referenced protocol [40]. The membrane was treated with Diaminobenzidine (DAB) until the brown colored bands were developed.

### MMP 2/9 zymographic analysis in breast cancer and surrounding tissues

The cytosols from tumor and its corresponding surrounding tissue were assayed for MMP activity. One percent type B-gelatin solution prepared in water, 8% resolving gel is prepared containing 1% B-gelatin solution. Samples were prepared in MMP dye and loaded in gel to run at 110 V for 60 minutes. Finally, the gel was stained in amide black or Coomasie blue solution for 1 hour at room temperature in a shaker. The gels were then destained with solution I for 15 to 30 minutes, followed by destaining solution II for 3 to 5 hours until clear MMP bands appeared. After washing the destaining solution the gel was incubated in a gel preservative solution for 15 minutes and finally scanned with a digital scanner [41].

### Determination of SULT1A1 activity in human tissues by PNPS assay method

The β-Naphthol sulfation activity was determined from the tumor tissue and its surrounding. This assay determines phenol sulfation activities of different isoforms of phenol sulphating SULTs. Tumor and surrounding tissue cytosols (50 μg protein) were used as the enzyme source in a total reaction volume of 250 μl. After 30 min incubation at 37ºC in a shaking water bath, the reaction was stopped by adding 250 μl of 0.25 M Tris, pH 8.7. The reaction mixtures were read at 401 nm in a spectrophotometer. Specific activity (SA) was expressed as nmole/minute/mg of protein [42].

### RT-PCR of SULT1E1 from rat hepatocytes

The RNA was isolated from hepatocyte of rats treated with DAS, Chalcone or their combination for 24, 48 and 72 hours. 1 μg of whole RNA utilized to perform reverse transcription PCR using Qiagen one step RT-PCR kit and SULT1E1 primer following the instructions provided in the kit. Total RNA concentration was measured by a NanoDrop Microvolume Spectrophotometers at 260 nm (A260 reading = 40 μg/ml RNA) and its purity was tested from the ratio of A260/A280. The master mix was prepared with stipulated proportions of ingredients including enzymes, dNTPs, template RNA and gene specific primers (see the Table at the end of this paragraph). Primer specificity was satisfactory, that was tested by universal nucleotide alignment). To compare with control, 500 bp cDNA of rat β-actin and 200 bp cDNA of human β-actin were synthesized and loaded in the gel (see below). Primers were designed using the GeneFisher primer designing software and published earlier [40, 42]. PCR was carried out in Eppendorf^®^ Mastercycler^®^ and the reaction program included reverse transcription at 50ºC for 30 min, PCR activation step 95ºC for 15 min, denaturation 94ºC for 1 min, annealing 64ºC for 1 min and extension at 72ºC for 1 min (30 cycles). Final extension was allowed for 10 min at 72ºC. PCR products were run an agarose gel electrophoresis image was captured by a BioRad gel doc system.

**Table:** 

Name of the gene studied	Forward primer	Reverse primer
Human and rat SULT1E1	5′-CTTCCAGTATCA TTTTGGGAAAAG-3′	5′-TGGATTGTTCTT CATCTC-3′
Rat β-actin	5′-GATGTACGTAGC CATCCA-3′	5′-GTGCCAACCAG ACAGCA-3′
Human β-actin	5′-GGCGGCAACAC CATGTACCCT-3′	5′-AGGGGA GGGA CTCGTCATACT-3′

### Activities assay of SOD, catalase, GPx and LDH activities by gel zymogram

#### Super oxide dismutase

Three different SOD activities were assayed, first in breast tumor and corresponding surrounding tissue, second in the serum of animal model treated with DAS, Chalcone and DAS + Chalcone for 7 days, 24, 48 and 72 hours and thirdly in the *in vitro* treated hepatocytes (3, 5 and 12 hour). A tablet of nitro blue tetrazolium (NBT) dissolved in 30 ml of water and the non-denaturing (10%) acrylamide gel kept soaked in it for 30 min followed by 40 ml SOD solution containing 0.028 M tetramethyl lethylenediamine (TEMED), 2.8 × 10^−5^ M riboflavin, and 0.036 M potassium phosphate at pH 7.8) for 15 min in shaking condition. Finally, the gel was placed on clean acetate sheet and illuminated for 5 to 15 mins. on a UV illuminator [43].

#### Catalase

A non-denaturing gel (8%) was loaded with a 25 μg of cytosol (from *in-vitro* treated rat hepatocytes for 3, 5 and 12 hours with DAS, Chalcone and DAS + Chalcone) and electrophoresed for 3 h at 40 mA at 4°C. The gel was washed 3 × 10 min in distilled water and incubated in 0.003% H_2_O_2_ for 10 minutes, followed by staining with 2% ferric chloride and 2% potassium ferricyanide. After rinsing with distilled water, the gel was scanned when the maximum contrast between the band and the background was obtained [43].

#### Glutathione peroxidase

A non-denaturing gel (8%) was loaded with a 150 μg of protein obtained from rat hepatocytes treated for different times with DAS and/or Chalcone. Electrophoresis carried out for 3 h at 40 mA at 4°C. The cumene hydroperoxides instead of H_2_O_2_ performed as a good substrate and the GPx (contains a selenium in the active site) can utilize this hydroperoxide to determine total peroxidase activity according to the established protocol as described in the referenced article [43].

#### Lactate dehydrogenase (LDH)

LDH zymogram in tumor and its corresponding surrounding tissue obtained from breast cancer patients was performed by using a standard protocol [44] with slight modification. LDH zymograms were obtained by separating 10 mg of protein on a native PAGE gel of 8% for 2 h at 4 W, then soaked in water followed by washing twice with water. Enzyme activity bands were developed from the conversion of NAD1 to NADH, which were clearly visible. The gel was scanned under the gel doc system.

### Determination of oxidative status and cytotoxicity parameters in rat hepatocytes

#### Estimation of malondialdehyde (MDA) levels

The MDA was estimated both from liver tissue and serum samples. Tissue was homogenized (10% w/v) in the ice-cold phosphate buffer (0.1 mol /L, pH 7.4) and the homogenate was centrifuged at 10,000 rpm at 4ºC for 10 min. The MDA assay was conducted using the supernatant following the protocol [45]. Finally, the MDA was measured and calculated utilizing the molar extinction coefficient of MDA (1.56 × 105 cm^2^/mmol).

### Estimation of non-protein soluble thiol (NPSH)

The NPSH in serum and liver tissue homogenates (prepared in 0.1 M phosphate buffer, pH 7.4) were determined by the standard DTNB (5, 5′- dithiobis-2-nitrobenzoic acid) method with a slight modification [46]. In brief, the protein was precipitated by trichloroacetic acid and clear cytosol was added to 0.1 M sodium phosphate buffer containing 5 μM DTNB. The level of NPSH was determined against a GSH standard curve.

### Evaluation of general toxicity

Serum glutamate pyruvate transaminase (SGPT), serum glutamate oxaloacetate transaminase (SGOT), NPSH and MDA were measured from the rat serum treated with DAS, Chal, and DAS + Chalcone for 24, 48 and 72 hours. SGPT and SGOT were measured by standard protocol with the assay kits (Ranbaxy, India or other reputed company). Total protein from serum was measured following the standard protocol [47].

### Histoarchitecture studies of cancer tissues

Breast tumor tissue and its corresponding surrounding tissues were embedded in paraffin, serially sectioned at 5 μM by an automated cryostat slicing machine (Leica Biosystems), stained with eosin and hematoxylin (Harris), and observed under a microscope (Nikon, Eclipse LV100, magnification 20X) to study the tissue histoarchitecture.

### Immunohistochemistry for SULT1E1

Tumor and its corresponding surrounding tissues were embedded in paraffin, serially sectioned at 5 μM by an automated cryostat slicing machine (Leica Bio systems). Sections were deparaffinized by baking at 60 c followed by xylene treatment, downgraded alcohol and water. Slides were washed with PBST containing 1% casein for 10 minutes, tissue sections were incubated with 5% casein for 30 minutes for preventing non-specific binding followed by overnight incubation in primary antibody SULT1E1 in 1% casein PBST, washed with 1% PBST and incubated in 1% casein PBST containing secondary antibody for 1 hour, washed with 1% PBST followed by water and stained with chromogenic substrate DAB for 3 minutes and then washed with water. Slides were fixed with mounting medium and observed under a microscope (Nikon, Eclipse LV100, magnification 20X) to study the SULT1E1 expression and localization.

### Statistical analysis

For the statistical evaluations of various factors, the SPSS for Windows statistical software suite (SPSS Inc., Chicago, IL, USA, 2016) was employed. Data from all parameters were collected from multiple independent set as well as replicated from a single set of experiments. The Kolmogorov-Smirnov test was used to examine the data distribution pattern. Students *t*’-test analysis and multiple comparison ANOVA tests were used to evaluate the baseline continuous variables and the results of the analyses. The level of significance was denoted within the 95% confidence limit and represented as *p* < 0.001 to *p* < 0.05.

### Data availability

Data are available upon request.

## CONCLUSIONS

The current study explores the events of the redox dependent adverse regulations of SULT1E1 and possible foul-play of estradiol in breast cancer tissues of postmenopausal women. This has been linked to the induction of HIF1α and MMP2/9 activation. In an attempt towards the therapeutic approach, we have demonstrated that Dialyl-sulfide is a good inducer of SULT1E1 gene and protein which significantly decreased the HIF1α and MMPs. In addition, chalcone played an ideal rectifier role of cellular redox environment. Together, the two drugs may be applied as effective therapeutic materials against some forms of breast cancer. To make definitive statements, proper pharmacological testing must be performed.

## SUPPLEMENTARY MATERIALS


