# Supplementary material for: Dialyl-sulfide with trans-chalcone prevent breast cancer prohibiting SULT1E1 malregulations and oxidant-stress induced HIF1a-MMPs induction
Source: Genes Cancer. 2024 Aug 9;15:41–59. doi: 10.18632/genesandcancer.237 (PMC11315411; doi:10.18632/genesandcancer.237)
Supplement: Supplementary file 1 [file ganc-15-237-s001.pdf]

Dialyl-sulfide with trans-chalcone prevent breast cancer prohibiting SULT1E1 malregulations and oxidant-stress induced HIF1a-MMPs induction

SUPPLEMENTARY MATERIALS

Supplementary Table 1: Details of the breast cancer patients. (Nazmeen et al., Cancer Cell Int. 2020; 20:70. Published 2020 Mar 4. https://doi.org/10.1186/s12935-020-1153-y).

SN	Age	Livelihood status	Nutritional status	Cancer grade	Disease description
1	60	Rural		IIIB	T4-65mm; N2-6 ALN; M0-no metastasis
2	45	Rural		IIIB	T4-57mm; N1-3 ALN; M0-no metastasis
3	50	Rural		IV	T4-74mm; N3-11 ALN; M0-Brain, liver, lung and bones
4	40	Rural		IIIA	T2-33mm; N2-6 ALN; M0- no metastasis
5	60	Rural		IIIB	T4-68mm; N1-3 ALN; M0-no metastasis
6	45	Rural		IIIB	T4-59mm; N1-2 ALN; M0-no metastasis
7	42	Rural		IIIB	T4-61mm; N1-2 ALN; M0-no metastasis
8	45	Rural	Ethnic food	IV	T4-71mm; N3-10 ALN; M0-no metastasis
9	40	Rural	Conserved food	IIIA	T2-41MM; N2-5 ALN; M0-no metastasis
10	40	Rural		IIIA	T2-44MM; N2-5 ALN; M0-no metastasis
11	40	Rural	Less fast food	IIIB	T4-62; N2-5 ALN; M0- Spread to chest wall
12	38	Rural		IIB	T2-22mm; N1- 2ALN; M0-no metastasis
13	45	Rural	Diet with less protein and more carbohydrate	IIIB	T4-70mm; N2- 8 ALN; M0-no metastasis
14	48	Semi-urban		IIIB	T4-66mm; N2- 7 ALN; M0-no metastasis
15	60	Rural	Low socio-economic status	IIIB	T4-78mm; N2- 8 ALN; M0-no metastasis, Swelling and Ulceration
16	45	Rural		IIIB	T4-73mm; N2- 9 ALN; M0-no metastasis
17	45	Rural		IV	T4-80mm; N3- 12 ALN; M0-Brain, liver, lungs
18	40	Rural		IIIA	T1-18mm; N0- No ALN; M0-no metastasis
19	40	Semi-urban		IIA	T1- 15mm; N0- No ALN; M0-no metastasis
20	27	Rural		IIIA	T2-39mm; N1- 2 ALN; M0-no metastasis
21	50	Rural		IIIA	T2-32mm; N1- 2 ALN; M0-no metastasis
22	38	Rural		IIB	T1- 20mm; N1- 1 ALN; M0-no metastasis
23	45	Rural		IIIB	T4-58mm; N2- 5 ALN; M0-no metastasis

Abbreviations: T: Tumor; N: Lymph Nodes; ALN: Axillary Lymph Nodes; M: Metastasis.
